# Scope and limitations of the preparation of xanthones using Eaton’s reagent

**DOI:** 10.55730/1300-0527.3624

**Published:** 2023-09-26

**Authors:** Johann BOSSON

**Affiliations:** Chemistry Department, Faculty of Sciences, Koç University, İstanbul, Turkiye

**Keywords:** Xanthone, Eaton’s reagent, condensation reaction, salicylic acid, phenol, phloroglucinol

## Abstract

Xanthones comprise a large family of heterocycles displaying fascinating biological properties. Many synthetic protocols have been developed for the preparation of natural and nonnatural xanthone derivatives. Among them, condensation reactions between salicylic acid derivatives and phenol partners are highly desirable. Those reactions can be satisfactorily performed using Eaton’s reagent (P_2_O_5_ in CH_3_SO_3_H). Despite being highly effective with a variety of substrates, this approach presents limitations that depend on the electronic nature of the reaction precursors. The scope and limitations of the Eaton’s reagent-mediated preparation of xanthones are herein presented and discussed. In short, this approach is limited to the utilization of very electron-rich phenol substrates (like phloroglucinol compounds), or to electron-rich phenol precursors (like resorcinol derivatives) via the isolation of benzophenone intermediates in this latter case. Electron-poor phenols are not amenable to this transformation with Eaton’s reagent.

## 1. Introduction

**Figure 1:**
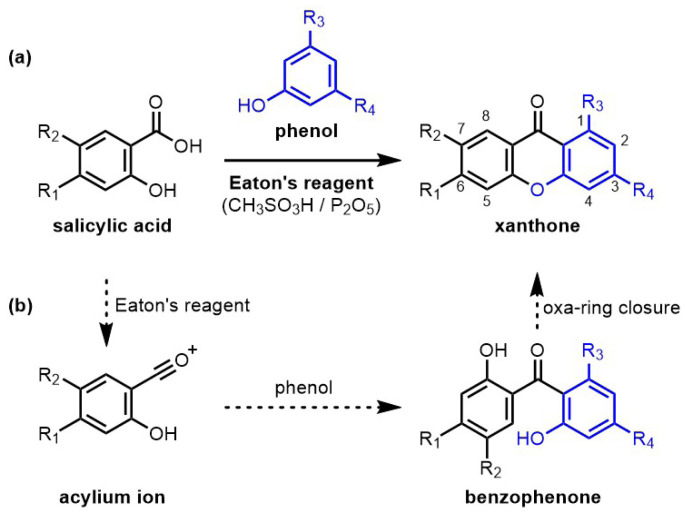
a): Synthetic approach toward xanthones by condensation reactions of salicylic acid and phenol precursors using Eaton’s reagent, and position numbering in the xanthone products; b): proposed reaction mechanism and intermediates.

Xanthones (Figure 1) are privileged heterocyclic structures highly sought after for their wide range of biological properties [1]. Xanthones exhibiting notably antibacterial [2], antioxidant [3], or antitumor properties [4] have been described. The xanthone skeleton is amenable to regioselective late-stage functionalization reactions, with this rich chemistry enabling the preparation of tailored derivatives for a myriad of biological targets. Furthermore, many (polyfunctionalized) xanthones are naturally occurring derivatives that can be isolated from fungi, lichens, and bacteria, among others [5,6].

In terms of synthesis and following the initial report by Michael [7], many approaches toward the xanthone scaffold were developed over the years targeting natural and nonnatural analogues with superior biological activities [8,9]. The chemistry of xanthones has been largely covered by several review articles [10–13], and the comprehensive compendium by Sousa et al. regarding developments from 2012 to 2020 is highly recommended [14]. In short, condensation reactions of salicylic acids with phenol derivatives, of aryl aldehydes with phenols, of 2-hydroxybenzaldehyde derivatives with 1,2-dihaloarenes via nucleophilic aromatic substitution reaction (S_N_Ar), or of 2-halobenzoic acid derivatives with in situ generated aryne partners are the most popular synthetic routes.

Condensation reactions between salicylic acid derivatives and phenols (Figure 1a) are particularly appealing due to the low cost of the reacting precursors, the atom-economy of the transformation (only two water molecules released), and the ease of the reaction protocols. In a seminal work, Grover et al. reported the use of zinc chloride (ZnCl_2_) in hot phosphoryl chloride (POCl_3_) to promote the reaction [15]. This methodology has been widely used despite some limitations [10,11]. Eaton’s reagent (7.7 wt.% of phosphorus pentoxide (P_2_O_5_) in methanesulfonic acid (CH_3_SO_3_H)) has been proposed in place of the ZnCl_2_/POCl_3_ mixture to perform this condensation reaction [16] and revealed superior activity [10,11]. Eaton’s reagent is known for its efficiency in acylation reactions [17]. In the context of the synthesis of xanthone derivatives, Eaton’s reagent promotes the formation of an acylium ion from the salicylic acid derivative (Figure 1b), which afterwards reacts with the phenol counterpart in a Friedel–Craft acylation reaction. The oxa-ring closure of the benzophenone intermediate (not isolated) happens next, providing the targeted product.

Despite being highly effective for a variety of substrates, the Eaton’s reagent-mediated condensation between salicylic acids and phenols presents limitations that are highly dependent on the electronic nature of the substrates. The scope and limitations of this approach are herein presented and discussed, with the aim to provide directions in the design and selection of substrates.

## 2. Materials and methods

### 2.1. General considerations

All commercial chemicals were purchased from Sigma Aldrich and were used as received. The reactions were monitored by thin-layer chromatography (TLC) carried out on silica gel plates using UV light as visualizing agent. Column chromatography was performed with silica gel (spherical, particle size 40 μm). NMR spectra and high-resolution mass spectroscopy (HMRS) were recorded at the n^2^STAR facility (Koç University). The ^1^H (500 MHz) and ^13^C (125 MHz) NMR spectra were recorded on a Bruker Avance III Ultrashield (500 MHz) spectrometer and were analyzed using Topspin. NMR chemical shifts are reported in parts per million with the solvent resonance as the internal standard (CDCl_3_, ^1^H: δ 7.26 ppm, ^13^C: δ 77.16 ppm; DMSO *d^6^*, ^1^H: δ 2.50 ppm, ^13^C: δ 39.52 ppm). Coupling constants (*J*) are reported in Hertz (Hz). Multiplicities are reported using the following abbreviations: s = singlet, brs = broad singlet, d = doublet, dd = double doublet, ddd = double double doublet, dt = double triplet, t = triplet, m = multiplet. The HMRS analyses were performed on a Waters Vion QTOF mass spectrometer.

### 2.2. Representative procedure of xanthone synthesis

Preparation of 1,3-dimethoxy-xanthone **5**: salicylic acid (2.07 g, 15.0 mmol, 1.5 equiv) and 1,3,5-trimethoxybenzene (1.68 g, 10.0 mmol, 1.0 equiv) were charged in a Schlenk tube under Ar atmosphere. Eaton’s reagent (10 mL) was added to the mixture and the Schlenk tube was sealed. The resulting slurry was stirred at 80 °C for 1 h 30 min, providing a dark brown solution at the end of the reaction. After cooling to ca. 25 °C, the reaction mixture was poured into ice, resulting in a pale pinkish slurry. This mixture was vigorously stirred for 20 min. The off-white (slightly pinkish) precipitate was collected by filtration and triturated with water (3 times). The material was then dried under a flow of air for 2 h. The solid residue was triturated with an Et_2_O/pentane (1:1) mixture (3 times) and the resulting light pink powder was dried under reduced pressure, giving 2.33 g (9.1 mmol) of **5** (yield: 91%).

### 2.3. Other synthetic procedures

Preparation of 3,6-dihydroxyxanthone **8**: 2,2′,4,4′-tetrahydroxybenzophenone **7** (730 mg, 2.96 mmol) was suspended in 6 mL of distilled water in an autoclave. The autoclave was sealed and heated at 200 °C for 24 h and then allowed to cool down to 20 °C. A brownish solid was collected from the reaction mixture by filtration. The solid residue was triturated in hot water (60 °C, 3 times) and in cold hexane (0 °C, 3 times). The resulting light brown solid was dried under a flow of air, affording 485 mg (2.13 mmol) of **8** as a light brown powder (yield: 72%).

Preparation of 3-methoxy-6-hydroxyxanthone **10**: the mixture of benzophenones obtained from the reaction of 1,3-dimethoxybenzene (1.32 mL, 10.0 mmol, 1 equiv) and 4-hydroxysalicylic acid (2.31 g, 15.0 mmol, 1.5 equiv) in Eaton’s reagent (see procedure above) was dissolved in CH_3_OH (50 mL). Then 2 M aq. NaOH solution (50 mL) was added to the reaction mixture, which turned into a yellow solution. The reaction mixture was refluxed for 6 h. After this time, the reaction mixture was cooled to 0 °C and was acidified with 2 M aq. HCl (60 mL), resulting in the formation of a white precipitate. The mixture was stored overnight at 4 °C to enhance precipitation. The precipitate was collected by filtration and triturated with water (3 times) and then with a mixture of acetone and chloroform (1:1, 3 times). The resulting material was dried under vacuum, affording 1.36 g (5.62 mmol) of **10** as an off-white solid (56% overall yield for two steps).

### 2.4. Characterizations of xanthone products and benzophenone intermediates

1,3-Dihydroxyxanthone **1**: **R***_f_* (cyclohexane/EtOAc 80:20, SiO_2_): 0.28. **^1^H NMR (500 MHz, DMSO *d****^6^***) δ** 12.82 (s, 1H), 11.13 (s, 1H), 8.12 (d, *J* = 7.4 Hz, 1H), 7.85 (t, *J* = 7.6 Hz, 1H), 7.59 (d, *J* = 8.2 Hz, 1H), 7.46 (t, *J* = 7.1 Hz, 1H), 6.40 (s, 1H), 6.22 (s, 1H). **^13^C NMR (126 MHz, DMSO *d****^6^***) δ** 179.8 (C), 166.0 (C), 162.9 (C), 157.5 (C), 155.4 (C), 135.7 (C*H*), 125.3 (C*H*), 124.5 (C*H*), 119.9 (C), 117.8 (C*H*), 102.3 (C), 98.2 (C*H*), 94.1 (C*H*). **HRMS (ESI, CH_3_OH)** calc. for C_13_H_8_O_4_: 228.0423; found: 228.0427. Data consistent with reported literature [18].

7-Nitro-1,3-dihydroxyxanthone **2**: **R***_f_* (CH_2_Cl_2_/CH_3_OH 95:05, SiO_2_): 0.44. **^1^H NMR (500 MHz, DMSO *d****^6^***) δ** 11.88 (brs, 2H), 8.77 (brs, 1H), 8.39 (brs, 1H), 7.45 (brs, 1H), 7.23 (brs, 2H). **^13^C NMR (126 MHz, DMSO *d****^6^*, only 7 peaks observed**) δ** 164.8 (C), 151.5 (C), 139.6 (C), 130.6 (C*H*), 128.3 (C*H*), 119.1 (C*H*), 115.9 (C). **HRMS (ESI, CH_3_OH)** calc. for C_13_H_7_NO_6_: 273.0273; found: 273.0278. For NMR characterization in acetone *d^6^*, see ref [19].

7-Bromo-1,3-dihydroxyxanthone **3**: **R***_f_* (cyclohexane/EtOAc 80:20, SiO_2_): 0.56. **^1^H NMR (500 MHz, DMSO *d****^6^***) δ** 12.57 (brs, 1H), 11.22 (brs, 1H), 8.18 (s, 1H), 8.00 (d, *J* = 7.7 Hz, 1H), 7.59 (d, *J* = 8.4, 1H), 6.42 (s, 1H), 6.24 (s, 1H). **^13^C NMR (126 MHz, DMSO *d****^6^***) δ** 178.5 (C), 166.3 (C), 162.8 (C), 157.3 (C), 154.4 (C), 138.1 (C*H*), 127.2 (C*H*), 121.5 (C), 120.4 (C*H*), 116.4 (C), 102.2 (C), 98.4 (C*H*), 94.3 (C*H*). **HRMS (ESI, CH_3_OH)** calc. for C_13_H_7_BrO_4_: 305.9528; found: 305.9531. Data consistent with reported literature [20].

1,3,6-Trihydroxyxanthone **4**: **R***_f_* (CH_2_Cl_2_/CH_3_OH 95:05, SiO_2_): 0.61. **^1^H NMR (500 MHz, DMSO *d****^6^***) δ** 13.05 (brs, 1H), 11.04 (brs, 1H), 10.96 (brs, 1H), 7.96 (brs, 1H), 6.88 (brs, 1H), 6.81 (brs, 1H), 6.34 (brs, 1H), 6.16 (brs, 1H). **^13^C NMR (126 MHz, DMSO *d****^6^***) δ** 179.1 (C), 165.2 (C), 164.3 (C), 162.9 (C), 157.5 (C), 157.4 (C), 127.2 (C*H*), 114.1 (C*H*), 112.3 (C), 102.1 (C*H*), 101.7 (C), 98.0 (C*H*), 94.0 (C*H*). **HRMS (ESI, CH_3_OH)** calc. for C_13_H_8_O_5_: 244.0372; found: 244.0370. Data consistent with reported literature [20].

1,3-Dimethoxyxanthone **5**: R*_f_* (cyclohexane/EtOAc 50:50, SiO_2_): 0.45. **^1^H NMR (500 MHz, CDCl_3_) δ** 8.27 (dd, *J* = 7.9, 1.6 Hz, 1H), 7.60 (ddd, *J* = 13.8, 8.5, 2.8 Hz, 1H), 7.33 (d, *J* = 8.3 Hz, 1H), 7.39 (dt, *J* = 15.8, 7.1 Hz, 1H), 6.46 (d, *J* = 2.3 Hz, 1H), 6.31 (d, *J* = 2.3 Hz, 1H), 3.96 (s, 3H), 3.88 (s, 3H). **^13^C NMR (126 MHz, CDCl_3_) δ** 175.5 (C), 165.0 (C), 162.1 (C), 159.9 (C), 155.0 (C), 133.8 (C*H*), 126.8 (C*H*), 123.9 (C*H*), 123.2 (C), 117.0 (C*H*), 95.2 (C*H*), 92.9 (C*H*), 56.4 (C*H_3_*), 55.8 (C*H_3_*). **HRMS (ESI, CH_3_OH)** calc. for C_15_H_12_O_4_: 256.0736; found: 256.0734. Data consistent with reported literature [21].

1,3-Dimethoxy-benzoxanthone **6**: **R***_f_* (SiO_2_, cyclohexane/EtOAc 60:40): = 0.35. **^1^H NMR (500 MHz, CDCl_3_) δ** 8.87 (s, 1H), 8.06 (d, *J* = 8.3 Hz, 1H), 7.88 (d, *J* = 7.9 Hz, 1H), 7.77 (s, 1H), 7.58 (t, *J* = 7.5 Hz, 1H), 7.48 (t, *J* = 7.4 Hz, 1H), 6.53 (d, *J* = 2.1 Hz, 1H), 6.35 (d, *J* = 2.0 Hz, 1H), 4.01 (s, 3H), 3.94 (s, 3H). **^13^C NMR (126 MHz, CDCl_3_) δ** 180.4 (C), 165.6 (C), 162.5 (C), 159.9 (C), 159.1 (C), 136.3 (C), 129.9 (C), 129.9 (C*H*), 128.7 (C*H*), 128.4 (C*H*), 127.1 (C*H*), 125.4 (C*H*), 122.8 (C), 115.9 (C*H*), 94.9 (C*H*), 93.1 (C*H*), 56.6 (C*H_3_*), 56.0 (C*H_3_*). **HRMS (ESI, CH_3_OH)** calc. for C_19_H_14_O_4_: 306.0892; found: 306.0893.

2,2′,4,4′-Tetrahydroxybenzophenone **7**: **R***_f_* (cyclohexane/EtOAc 50:50, SiO_2_): 0.22. **^1^H NMR (500 MHz, DMSO *d****^6^***) δ** 11.26 (brs, 2H), 10.24 (brs, 2H), 7.18 (d, *J* = 8.9 Hz, 2H), 6.32 (brs, 4H). **^13^C NMR (126 MHz, DMSO *d****^6^***) δ** 199.3 (C), 162.8 (C), 160.6 (C*H*), 133.5 (C), 115.2 (C), 107.3 (C*H*), 102.4 (C*H*). **HRMS (ESI, CH_3_OH)** calc. for C_13_H_10_O_5_: 246.0528; found: 246.0526. Data consistent with those reported in the Spectral Database for Organic Compounds (SDBS) under the reference SDBS No. 1488 by the National Institute of Advanced Industrial Science and Technology (AIST).

3,6-Dihydroxyxanthone **8**: **R***_f_* (CH_2_Cl_2_/CH_3_OH 95:05, SiO_2_): 0.20. **^1^H NMR (500 MHz, DMSO *d****^6^***) δ** 10.81 (brs, 2H), 7.98 (d, *J* = 7.5 Hz, 2H), 6.86 (d, *J* = 7.2 Hz, 2H), 6.82 (brs, 2H). **^13^C NMR (126 MHz, DMSO *d****^6^***) δ** 173.9 (C), 163.4 (C), 157.5 (C), 127.8 (C*H*), 114.0 (C), 113.7 (C*H*), 102.1 (C*H*). **HRMS (ESI, CH_3_OH)** calc. for C_13_H_8_O_4_: 228.0423; found: 228.0424. Data consistent with reported literature [22].

3-Hydroxy-6-methoxyxanthone **10**: **R***_f_* (CH_2_Cl_2_/acetone 80:20, SiO_2_): 0.61. **^1^H NMR (500 MHz, DMSO *d****^6^***) δ** 10.91 (brs, 1H), 8.05 (d, *J* = 8.8 Hz, 1H), 8.01 (d, *J* = 8.7 Hz, 1H), 7.11 (d, *J* = 2.3 Hz, 1H), 7.01 (dd, *J* = 8.8, 2.3 Hz, 1H), 6.89 (dd, *J* = 8.7, 2.2 Hz, 1H), 6.85 (d, *J* = 2.1 Hz, 1H), 3.91 (s, 3H). **^13^C NMR (126 MHz, DMSO *d****^6^***) δ** 174.0 (C), 164.4 (C), 163.7 (C), 157.6 (C), 157.5 (C), 127.8 (C*H*), 127.4 (C*H*), 115.0 (C), 113.9 (C*H*), 113.2 (C*H*), 102.1 (C*H*), 100.6 (C*H*), 56.1 (C*H_3_*). **HRMS (ESI, CH_3_OH)** calc. for C_14_H_10_O_4_: 242.0579; found: 242.0581. Data consistent with reported literature [23].

## 3. Results

The electronic parameters governing the efficacy of the preparation of xanthone derivatives by condensation reactions of salicylic acid precursors and phenol derivatives using Eaton’s reagent were investigated using diversely functionalized substrates (Figure 1). As will be discussed in the following paragraphs, it appears that the electron richness of the phenolic reaction partners plays a crucial role in the reaction outcome.

### 3.1. Reactivity with phloroglucinol derivatives

**Figure 2:**
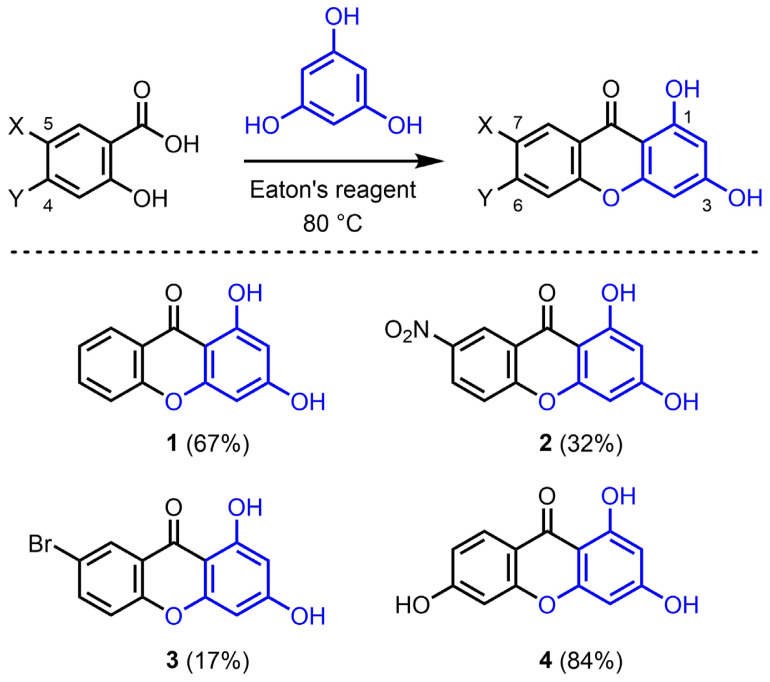
Preparation of 1,3-dihydroxyxanthones **1**–**4** by condensation of phloroglucinol and salicylic derivatives using Eaton’s reagent. Isolated yields are given in parentheses.

The reactivity of phloroglucinol (1,3,5-trihydroxybenzene) was investigated first (Figure 2). Phloroglucinol can be considered a highly electron-rich surrogate of phenol. Satisfactorily, treatment of a mixture of phloroglucinol and salicylic acid (1.5 equiv) performed in Eaton’s reagent at 80 °C for 1 h 30 min afforded 1,3-dihydroxyxanthone **1** in 67% isolated yield after simple precipitation in ice and trituration in a pentane/Et_2_O mixture. The influence of the electronic parameters of the salicylic acid derivatives was analyzed next. Electron-poor 5-nitrosalicylic acid provided 1,3-dihydroxy-7-nitroxanthone **2** in 32% yield. Similarly, 5-bromosalicylic acid provided 7-bromo-1,3-dihydroxyxanthone **3** in 17% isolated yield only. It was initially hypothesized that electron-poor salicylic acid derivatives would generate more electrophilic acylium intermediates and hence increase the reaction yields, but those highly reactive intermediates led to the formation of numerous side products. On the contrary, electron-rich 4-hydroxysalicylic acid afforded 1,3,6-trihydroxyxanthone **4** in 84% isolated yield. The higher yield obtained with this reagent was ascribed to the higher stability of the corresponding acylium intermediate.

**Figure 3:**
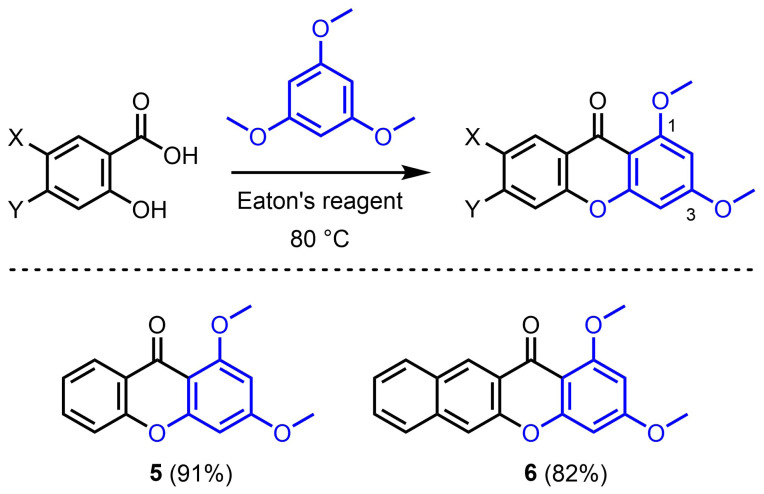
Preparation of 1,3-dimethoxyxanthones **5** and **6** by condensation of 1,3,5-trimethoxybenzene and salicylic derivatives using Eaton’s reagent. Isolated yields are given in parentheses.

The reactivity of 1,3,5-trimethoxybenzene was also briefly investigated (Figure 3). Pleasingly, its reaction with simple salicylic acid afforded the corresponding 1,3-dimethoxyxanthone **5** in 91% isolated yield. Condensation between 1,3,5-trimethoxybenzene and 3-hydroxy-2-naphthoic acid provided extended 1,3-dimethoxy-benzoxanthone **6** in 82% yield.

### 3.2. Reactivity with resorcinol derivatives

**Figure 4:**
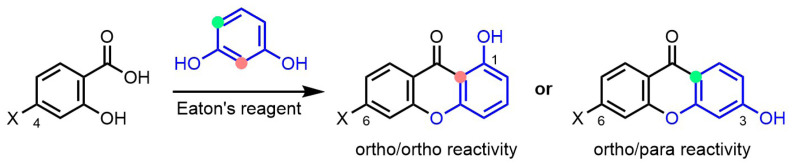
Possible regioisomers of xanthone products with resorcinol substrate. Orange: ortho/ortho position; green: ortho/para position.

Less electron-rich resorcinol (1,3-dihydroxybenzene) derivatives were next evaluated in the construction of xanthones using Eaton’s reagent. Importantly with this class of substrates, two reactive sites are formally possible for the acylation reaction, which would lead to the formation of two xanthone regioisomers (Figure 4). Reactivity from the position ortho/ortho relative to the two hydroxy groups of the resorcinol would yield xanthones functionalized at position 1, whereas reactions from the position ortho and para relative to the two hydroxy groups would afford products functionalized at position 3. To simplify the identification of the reaction products, 4-hydroxysalicylic acid was selected as reacting partner. Indeed, with this substrate, reactivity from the ortho/para position would lead to a symmetrical product that would be easily distinguished by NMR spectroscopy from the unsymmetrical xanthone resulting from reactivity of the ortho/ortho position. Furthermore, as discussed above and shown in Figure 2, the electron richness of 4-hydroxysalicylic acid was expected to favor the reaction.

**Figure 5:**
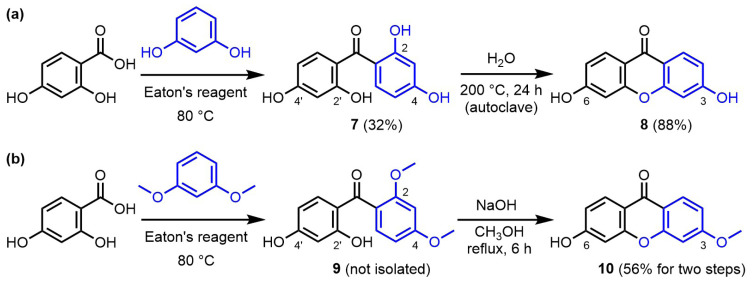
a): Two-step preparation of 3,6-dihydroxyxanthone **8** via benzophenone **7**; b) two-step preparation of 3-methoxy-6-hydroxyxanthone **10** via benzophenone **9**.

Experimentally, reaction of 4-hydroxysalicylic acid with resorcinol performed in Eaton’s reagent at 80 °C led rapidly to a mixture of products and it was observed that stopping the reaction after 40 min allowed the formation of side products to be limited. Precipitation/trituration revealed in this case was inefficient to afford a clean product, and column chromatography (SiO_2_, pentane/Et_2_O) had to be performed to isolate the main product of the reaction. ^1^H and ^13^C NMR spectroscopy confirmed the formation of a symmetrical compound (reactivity from the ortho/para position) matching the targeted xanthone product, but the chemical shift of the carbonyl group (~200 ppm) was drastically different from that observed with xanthones obtained from phloroglucinol (~175 ppm). As such, a symmetrical benzophenone product (virtually impossible to differentiate from the targeted xanthone product by NMR spectroscopy) was proposed to rationalize this observation, which was confirmed by mass spectroscopy. Overall, treatment of a mixture of 4-hydroxysalicylic acid and resorcinol with Eaton’s reagent afforded symmetrical 2,2′,4,4′-tetrahydroxybenzophenone **7** in 32% yield only (Figure 5, panel a). Satisfactorily, benzophenone **7** could be converted to the corresponding 3,6-dihydroxyxanthone **8** by simple thermolysis in water (200 °C in an autoclave, 24 h, 88% yield) [22].

An analogous approach was attempted using 1,3-dimethoxybenzene in place or resorcinol (Figure 5, panel b). Similarly, the Eaton’s reagent-mediated reaction stopped at the benzophenone step. However, in this case, an inseparable mixture of the two regioisomers resulting from the ortho/para and ortho/ortho reactivities in a 3:1 ratio was obtained, and benzophenone **9** could not be isolated. Nevertheless, treatment of the mixture of benzophenones with 2 M aq. NaOH in refluxing methanol for 6 h afforded the targeted 3-methoxy-6-hydroxyxanthone **10** in 56% overall yield for two steps [14,24].

### 3.3. Reactivity with phenol derivatives

**Figure 6:**
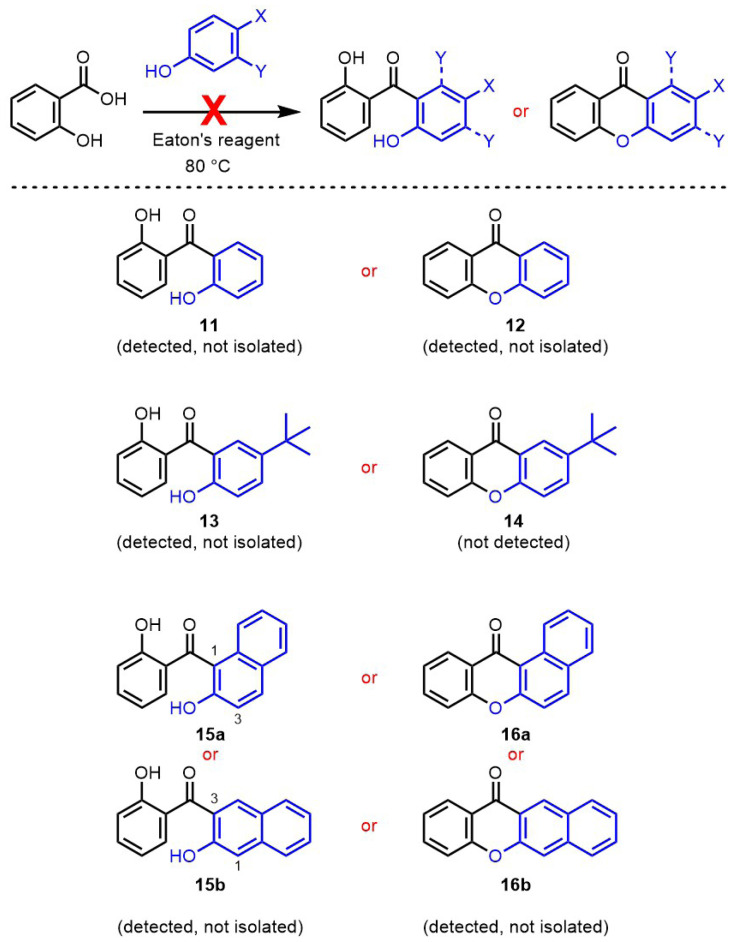
Attempted condensation reactions between salicylic acid and phenol derivatives using Eaton’s reagent.

Phenol derivatives were also investigated as substrates in condensation reactions with salicylic acid using Eaton’s reagent. Considering the results obtained with phloroglucinol and resorcinol substrates, xanthone products but also benzophenone intermediates were targeted (Figure 6). Reaction of simple phenol with unfunctionalized salicylic acid afforded an inextricable mixture of products, which could not be separated by crystallization, selective precipitation, or column chromatography. NMR spectroscopy could not confirm the formation of either the benzophenone intermediate **11** or the xanthone product **12**. However, **11** and **12** could be detected by mass spectrometry after column chromatography. It is noteworthy that the formation of an unproductive benzophenone resulting from the para-addition of phenol onto salicylic acid could not be ruled out with this spectroscopy technique. With more electron-rich para-tert-butylphenol, many products were formed but could not be separated, with most of the phenol substrate remaining unreacted. Traces of benzophenone **13** were detected by NMR spectroscopy and confirmed by mass spectroscopy, in contrast to xanthone **14**, which could not be identified in the mixture. Finally, the reactivity from positions 1 or 3 of 2-naphthol was considered, targeting benzophenones **15a** or **15b**, or extended xanthones **16a** or **16b**, respectively. With this substrate, full conversion of the naphthol was observed in the condensation reaction, but many reaction products were obtained. Unfortunately, their separation could not be achieved. Intermediate benzophenones of type **15** were clearly observed by NMR spectroscopy, but they could not be isolated and the regioselectivity toward products **15a** or **15b** could not be determined. Xanthones of type **16** were also detected by mass spectroscopy, but could not be confirmed by NMR analysis.

## 4. Discussion

The parameters governing the condensation reactions between salicylic acid derivatives and phenol substrates performed in Eaton’s reagent to yield xanthone products were scrutinized. It was evidenced that the electron richness of both the salicylic acid electrophiles and the phenol nucleophiles are key parameters. Rather counterintuitively, more electron-rich salicylic acid electrophiles gave better yields than their more electron-poor counterparts (Figure 2). This can be attributed to the formation of highly reactive acylium intermediates with these latter substrates, inducing the formation of many side products. The enhanced stability of the electron-rich acylium intermediates was favorable in this transformation.

**Figure 7:**
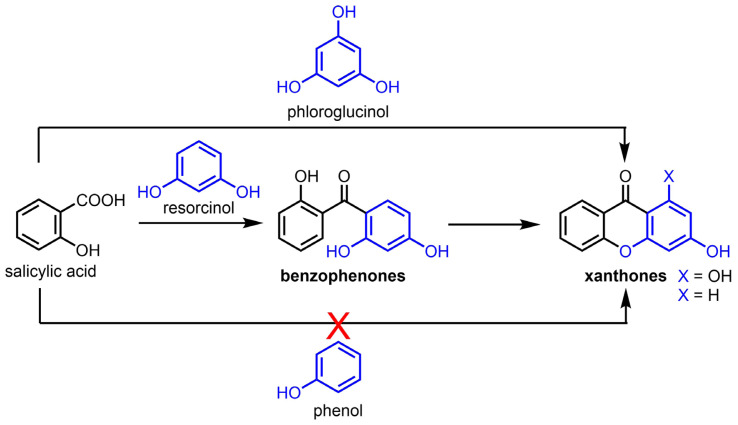
Impact of the electron richness of the phenol substrates on the Eaton’s reagent-mediated formation of xanthones by condensation with salicylic acid derivatives.

While the electron-richness of the salicylic acid derivatives markedly influences the reaction yields, the electron richness of the phenol substrates plays an even more critical role in the reaction outcome (Figure 7). Reactions with very electron-rich phloroglucinol substrates readily afforded the targeted xanthones (Figures 2 and 3). Electron-rich resorcinol analogues failed to provide the xanthone products, but allowed the isolation of benzophenone intermediates, which could be easily transformed into the oxa-ring closed species (Figure 5). With both phloroglucinol and resorcinol starting materials, it was observed that methoxy analogues (i.e. 1,3,5-trimethoxybenzene and 1,3-dimethoxybenzene, respectively) gave better yields. Conversely, simple phenol derivatives were inefficient in promoting the formation of either benzophenone intermediates or xanthone products (Figure 6).

Overall, the Eaton’s reagent-mediated preparation of xanthones is very attractive as it consists of a simple reaction protocol and it usually involves cheap commercially available starting materials. However, as this approach is limited to the utilization of electron-rich phenol substrates, careful selection of the reacting partners is required to achieve the synthesis of the targeted structures.
